# Supplementary material for: Modeling glioblastoma heterogeneity as a dynamic network of cell states
Source: Mol Syst Biol. 2021 Sep 16;17(9):e10105. doi: 10.15252/msb.202010105 (PMC8444284; doi:10.15252/msb.202010105)
Supplement: Supplementary file 6 — Source Data for Figure 5 [file MSB-17-e10105-s004.zip › Figure5A_sourcedata/GSEA_3071/hallmarks_stateVI.GseaPreranked.1623742557031/HALLMARK_SPERMATOGENESIS.html]

Details for gene set HALLMARK\_SPERMATOGENESIS[GSEA]

|  || Dataset | state63071 |
| Phenotype | NoPhenotypeAvailable |
| Upregulated in class | na\_neg |
| GeneSet | HALLMARK\_SPERMATOGENESIS |
| Enrichment Score (ES) | -0.36391306 |
| Normalized Enrichment Score (NES) | -1.3155264 |
| Nominal p-value | 0.17980637 |
| FDR q-value | 0.4034579 |
| FWER p-Value | 0.998 |
Table: GSEA Results Summary

  

Fig 1: Enrichment plot: HALLMARK\_SPERMATOGENESIS      
 Profile of the Running ES Score & Positions of GeneSet Members on the Rank Ordered List

  

| PROBE | GENE SYMBOL | GENE\_TITLE | RANK IN GENE LIST | RANK METRIC SCORE | RUNNING ES | CORE ENRICHMENT || 1 | NF2 |  |  | 68 | 0.817 | 0.0493 | No |
| 2 | PSMG1 |  |  | 246 | 0.568 | 0.0476 | No |
| 3 | AURKA |  |  | 572 | 0.432 | -0.0062 | No |
| 4 | ARL4A |  |  | 704 | 0.395 | -0.0095 | No |
| 5 | CCNB2 |  |  | 967 | 0.350 | -0.0528 | No |
| 6 | NEK2 |  |  | 1307 | 0.302 | -0.1214 | No |
| 7 | RFC4 |  |  | 2185 | -0.312 | -0.3379 | Yes |
| 8 | CAMK4 |  |  | 2189 | -0.313 | -0.3127 | Yes |
| 9 | TALDO1 |  |  | 2250 | -0.324 | -0.3023 | Yes |
| 10 | JAM3 |  |  | 2275 | -0.328 | -0.2816 | Yes |
| 11 | BRAF |  |  | 2294 | -0.332 | -0.2590 | Yes |
| 12 | STRBP |  |  | 2393 | -0.352 | -0.2567 | Yes |
| 13 | PEBP1 |  |  | 2463 | -0.369 | -0.2451 | Yes |
| 14 | NCAPH |  |  | 2471 | -0.370 | -0.2161 | Yes |
| 15 | GMCL1 |  |  | 2597 | -0.400 | -0.2174 | Yes |
| 16 | DCC |  |  | 2658 | -0.414 | -0.1995 | Yes |
| 17 | TOPBP1 |  |  | 2774 | -0.448 | -0.1940 | Yes |
| 18 | SPATA6 |  |  | 3143 | -0.614 | -0.2447 | Yes |
| 19 | GAD1 |  |  | 3266 | -0.726 | -0.2180 | Yes |
| 20 | EZH2 |  |  | 3282 | -0.741 | -0.1604 | Yes |
| 21 | CDK1 |  |  | 3396 | -0.883 | -0.1181 | Yes |
| 22 | PIAS2 |  |  | 3455 | -1.018 | -0.0494 | Yes |
| 23 | PCSK1N |  |  | 3522 | -1.197 | 0.0321 | Yes |
Table: GSEA details [plain text format]

  

Fig 2: HALLMARK\_SPERMATOGENESIS: Random ES distribution      
 Gene set null distribution of ES for **HALLMARK\_SPERMATOGENESIS**
